# Conference on Railway Car Sanitation; the New Texas Law

**Published:** 1903-08

**Authors:** 


### CONFERENCE ON RAILWAY CAR SANITATION; THE NEW TEXAS LAW.

Dr. Geo. R. Tabor, State Health Officer of Texas, asked the surgeons of the Texas railroads to meet him at Austin on the 4th inst. for the purpose of a general conference as to what is being done by the several roads in the way of sanitation, and the steps that should be taken to carry into effect the new law requiring disinfection of sleepers and day coaches. The law requires disinfection of railway coaches, under heavy penalty for failure, the manner and means and the time of such disinfection to be in accordance with rules and regulations to be prescribed by the State Health Officer. Before formulating said rules Dr. Tabor wished to get the views of the distinguished gentlemen who represent these important interests. Of course, when a car becomes infected with any of the acute contagious diseases, and is known to be infected, it is turned over to the quarantine officer at nearest point for fumigation and cleansing before it is again used. But the assumption is that upholstered cars, both day and sleeping, are infected all the time, with tubercle bacilli especially, and to cause the removal of this and other great dangers to the traveling public, as far as it is possible to remove it, is the end and object of the law, and of the conference. Texas is the Mecca to which thousands of persons afflicted with tuberculosis travel in hope of cure or betterment by the mild climate, and of course they infect the cars. The incoming of these unfortunates is greatest on the approach of winter, and the exodus occurs in the spring. The travel of consumptives on the Texas railroads is very heavy at those times. The general public must be protected from the poison given off by them. It is a difficult problem and can not be at once and entirely solved; but much can be done to lessen the evil.

Dr. Tabor's call was responded to by the following surgeons, representing the principal roads doing business in Texas:

Dr. G. W. Cale, Springfield, Mo., Chief Surgeon Frisco Line; Dr. S. C. Red, Houston, Texas, Chief Surgeon Houston and Texas Central; Dr. R. W. Knox, Houston, Texas, Chief Surgeon Southern Pacific (Sunset lines); Dr. Bacon Saunders, Fort Worth, Texas, Chief Surgeon Fort Worth and Denver Lines; Dr. W. A. Duringer, Fort Worth, Texas, Chief Surgeon Rock Island Line; Dr. C. A. Smith, Tyler, Texas, Chief Surgeon Cotton Belt Line; Dr. W. G. Jameson, Palestine, Texas, Chief Surgeon International and Great Northern System; Dr. W. C. Jones, Walnut Springs, Texas, Chief Surgeon Texas Central; Dr. W. H. Monday, Terrell, Texas, Chief Surgeon Texas Midland; Dr. A. C. Scott, Temple, Texas, Chief Surgeon Gulf, Colorado and Santa Fe; Dr. R. F. Miller, Sherman, Texas, Oculist to Frisco Line, Katy and others.

There were also present Dr. Jno. Preston, Superintendent of the Epileptic Colony, Abilene; Mr. D. H. Martyn, of St. Louis, Division Superintendent of the Pullman Sleeping Car Co.; Mr. O. S. Newell, of San Antonio, Division Superintendent of the Pullman Sleeping Car Co.; Mr. Ennis, of the same interest, and a distinguished and representative traveler of the Commercial Travelers or T. P. A. Association, in the person of Mr. Marchbank. The editor of this journal was also present by the courtesy of an invitation from the State Health Officer.

Dr. Tabor called on each of the gentlemen present for a statement of what sanitary measures are employed on their respective roads and for suggestions for the better protection of the traveling public, and each one responded, briefly detailing the orders which are given to their trainmen and other employes. It consisted of general sanitary policing, and in some instances of efforts at disinfection, by the use of solutions of bichloride of mercury in spittoons, the use of sawdust dampened with it for sweeping, its use as spray in some cases, the blowing out of cars by compressed hot air, etc.

Mr. Martin said that sheets wet with formaline were hung in the coaches at the end of the run, etc., in addition to blowing out and the use of a disinfectant, the formula of which he read.

Mr. Marchbank said he had traveled on every road in Texas, often riding in the second class or general coaches, where travelers smoke and spit on the floor and put their feet on the seats or in a fellow passenger's lap, and some times do the hog act generally, and to his certain knowledge the rules and regulations as to spitting, etc., and keeping clean generally, were not enforced or obeyed. There is a crying need for authority on the part of somebody to enforce these rules.

When all the representatives of railroad and traveling interests had been heard, Dr. Tabor said he wanted to hear from Dr. Daniel, the editor of the "Red-Back," one known to all present as deeply interested in sanitary science and the public health.

Dr. Daniel responded briefly. He said that all the reports showed more or less efficient sanitary policing, but that with the exception of the International and Great Northern Railroad none of the roads were disinfecting the cars in the sense contemplated by the law. Dr. Jameson, Chief Surgeon of that road, had said that the International and Great Northern were using, with satisfaction, a certain device of a formaldehyde generator. Dr. Daniel pointed out that the law requires disinfection, sterilization; and that this can not be accomplished by hot air, ventilation, sunshine, cleaning up, nor by the use of liquids as spray, nor by sheets wet with formaline. The danger lies in the dust of the car, in which the living organisms of consumption, coughed or spit up by consumptive travelers, and other diseases have found lodgment, and this dust permeates the carpets, curtains, cushions and all textile equipment in the car; they can not be reached by any other agency than a germicidal gas, which must be generated in such volume as to be forced into and through all such fabrics. It is the conclusion of all authorities, he said, that for this purpose formaldehyde gas alone is efficient. He pointed out the objections to sulphur, or chlorine, their bleaching and corroding properties. Besides these objections, they do not kill pathogenic bacteria. The choice of germicides is narrowed down to a gas, which alone can reach the bacilli anywhere and everywhere and kill them, and the choice of a gas is narrowed down to formaldehyde. There are numerous devices on the market for making formaldehyde—all more or less efficient—and it is only a matter of choice, he said, or selection of the device found by trial to generate the gas in greatest quantity and with the greatest rapidity, *cæteris paribus*.

So long as cars are upholstered with germ-harboring fabrics, so long would it be necessary to *fumigate* them; no amount of cleaning up or keeping clean will sterilize them. The day will come, he said, when the interest of the public health will demand that sleepers shall be as aseptic as a surgeon's operating room. It should contain nothing that can not be wiped off with a sponge wet with bi-chloride solution. Evolution is taking place in the sleeper as it did in the bedstead. We older ones remember when the bed and bedstead were an abomination in the sight of the Lord, with its curtains and things to harbor all sorts of germs. Now we have enameled iron or brass bedsteads, as light as a cane chair, and the thinnest mattress and woven wire springs.

"If I were a railroad surgeon," said he, "I would advise the rapid elimination of all equipment in cars that can harbor disease germs. As new cars are constructed, let them be equipped with rattan or woven wire seats, discard woolen curtains and blankets, throw away all carpets and rugs. Use linen or glazed canvas curtains if you must have curtains. They can be boiled or washed with germicide solutions. In a few years this new car will have replaced the death-dealing, disease-harboring abominations now in use, and in which the unsuspecting public have to sleep. A man takes his life in his hand every time he lies down in one of the stuffy, ill-ventilated, over-heated, dust and disease-infected 'berths,' so-called."

In conclusion, Dr. Tabor thanked the gentlemen for their presence and assistance and said that he could not tell just when the rules would be issued, as his office force is extremely limited. He assured the surgeons that while the law would be enforced he would not assume autocratic power; that within the next few days some regulations will be issued, which, he said, would meet with general approval. He would adopt the suggestions for experiments to find a practical germicide and said they would be conducted. Thought disinfection should precede sweeping so as to not throw germs out on the public; objected to sweeping en route and said he would be particular as to watering the public. He also said the day has come for the elimination of plush, hangings, etc. Blowing out the dust does not destroy the germs; it only unloads them on the outside public. Dr. Tabor gave his views at length. His remarks met with much approval and were greeted with applause.

---

Another yellow fever scare is on. If the disease is only propagated by the *steg. fac.* as the authorities say, it is only a matter of mosquito bars, and no guilty mosquito should be allowed to escape from Tampico or Merida. But the mosquito theory advocates—Surgeon General Wyman et al.—are not willing to put their faith to the touch; they depend still on quarantine and disinfection. So does Texas. It is sorter like the parson who, when his horse ran away with his buggy, trusted to providence till the breeching broke; then he jumped out.

